# Vaccine hesitancy as self-determination: an Israeli perspective

**DOI:** 10.1186/s13584-016-0071-x

**Published:** 2016-04-04

**Authors:** Baruch Velan

**Affiliations:** Gertner Institute for Epidemiology and Health Policy Research, Tel-Hashomer, Ramat Gan, Israel

## Abstract

Vaccine hesitancy can be portrayed as a broad spectrum of phenomena, ranging from a genuine call for help to complete defiance of authorities. The emphasis here is made on mid-spectrum hesitancy; hesitancy as an act of personal exploration and deliberation whether to get vaccinated or not. This form of vaccine hesitancy can be identified in the attitude of the Israeli public towards routine childhood vaccination programs, seasonal flu vaccination, newly introduced vaccines, such as human papilloma virus vaccine, as well as towards the emergency vaccination programs against poliovirus and H1N1 pandemic influenza. Vaccine hesitancy in Israel appears to be a process where individuals exercise self-determination and self-empowerment and make their own decisions based on assessment, reflection, choosing between various options and dealing with considerable complexities.

Addressing this form of vaccine hesitancy could be challenging, but ultimately fruitful. This would require change of attitudes on the part of policymakers. The first steps should involve the realization that deliberative hesitancy is here to stay, and that hesitant individuals should be respected. This could pave the way for designing appropriate intervention strategies for convincing the hesitant public about the advantages of vaccination.

## Defining vaccine hesitancy

Despite compelling evidence of the value of vaccines in preventing infectious diseases and in saving lives, in recent years vaccine hesitancy has become a growing focus of attention and concern. In the year 2015 alone, ~75 articles dealing with vaccine hesitancy were published, including more than 10 comprehensive reviews and meta-analyses (for a representative sample of these publications, please see references [1–5]). This abundance is not only a manifestation of the interest in the subject, but is also an indication of the prevalent confusion and discordance in the field. Questions are being asked about the definition of vaccine hesitancy, its manifestations, and the ways to measure them. This impedes the identification of targets for intervention, and development of appropriate measures to deal with hesitancy. Thus, the hesitancy field may appear to be over-expanding, but the discussion is far from being exhausted.

The review by Kumar et al., published in this journal [6] is a valuable contribution to the understanding of vaccine hesitancy. The authors use a very wide prism to describe the phenomenon, suggesting that hesitancy includes every manifestation between full acceptance of vaccines and outright refusal of all vaccines. Moreover, Kumar et al., claim that the causes of vaccine hesitancy can be described as a complex interaction of external, agent-specific and host-specific factors. By doing so, they bring into the equations factors such as immunization requirements, policies, media, norms, vaccine-efficacy, vaccine safety, race, education, income and knowledge about vaccines. They also indicate that hesitancy is complex and context dependent, varying across geographies, cultures and vaccine types. This very legitimate approach allows the readers to perceive the full complexity of the situation, and the varied obstacles that face policymakers. Nevertheless, very wide pictures often tend to be blurred. The very wide perspective used by Kumar et al., can obstruct effective intervention by health authorities: The target definition appears to be too wide for policymakers to deal with. One will need to defined priorities before designing strategies to counteract the effects of vaccine hesitancy, and these should be shaped to fit the specific features of hesitancy in a given scenario.

An alternative approach to addressing vaccine hesitancy, that is proposed here, is based on deviation from the broad definitions. This can be achieved by focusing on hesitancy in a very local, culture-dependent setting (the Israeli population) and by defining hesitancy more precisely, relating to it as a personal deliberation process of whether to get vaccinated or not.

## Mapping of vaccine hesitancy in Israel

Thematic analysis allows for the mapping of vaccine hesitancy in Israel into four major domains: Hesitancy related to routine vaccination programs; Hesitancy related to newly introduced vaccination programs; Hesitancy related to emergency vaccination; and hesitancy expressed by health care personnel (HCP) towards vaccination related to their professional duties. Several recent Israeli studies provide indications of these four manifestations of vaccine hesitancy, as demonstrated either by the behavior of target populations or by the attitudes expressed by lay individuals.

### Hesitancy related to routine childhood vaccination programs

In recent years, health care professionals have noted an increase in the number of Israeli parents who immunize their children, but do so to an extent, or at ages that differ from the officially recommended protocols (Personal communication by Bela Elran, chief epidemiology nurse, MOH). In two recent studies, attempts were made to quantify this phenomenon. In one study, based on self-reporting [7], the percentage of parents deviating from the recommended protocols amounted to ~9 %. In a different study, based on records from Mother-Child Health Clinics, the percentage of children who did not complete the recommended vaccination protocols was 7.5 % [8]. Interestingly, in both studies more than of half of the parents indicated that the deviations were a result of parental decision. When asked about the reason for such decisions, typical answers related to delaying vaccination, to spreading injections over more visits, or to omitting specific vaccines.

### Hesitancy related to seasonal flu vaccination programs

The debate around seasonal flu vaccination reemerges in Israel at the beginning of every winter. This annual ritual is fed by three major concerns related to the shifting nature of the influenza virus strains, to the annual variations in the disease severity, and to variations in the efficacy of influenza vaccine cocktails. These concerns appear to provide a solid ground for hesitancy related to flu vaccination, manifested by the undulating partial coverage in all target groups. The reported coverage rates are 57-64 % in the elderly, 25-42 % in the chronically ill and 20-25 % in children (ICDC records, curtsey of Tamar Shohat and Zalman Kaufman).

A recent study [9] on the attitude of Israelis to seasonal Influenza vaccination identified three different groups. The two groups which are easiest to understand are Acceptors (22 %), who will receive vaccination unconditionally, and Non-Acceptors (34 %), who will never get vaccinated. Alongside these two groups is a large group (44 %) defined as Conditional Acceptors. Members of this group will consider vaccination, if and when favorable measures (such as physician recommendation) are implemented. This group which exhibits an intermediate risk perception towards influenza infection can be easily defined as hesitant. One can claim that these individuals have not formulated as yet a concrete perception on the value of seasonal vaccination programs.

### Vaccination hesitancy related to an emergency polio vaccination campaign

In 2013, an outbreak of wild poliovirus was detected in a sewage sample in Israel where inactivated polio vaccine (IPV) had been used exclusively since 2005. In order to curtail the outbreak, a nationwide vaccination campaign using oral polio vaccine (OPV) was conducted, targeting all children under age ten [10]. This emergency polio vaccination campaign led to an animated public debate, which was conducted in the formal media, as well as on online platforms. During the month of August 2013 alone, a Web search identified 32,500 polio-related discussions [11]. Content analysis of these discussions [11, 12] revealed motives related to vaccine acceptance and vaccine rejection, but also clear manifestations of hesitancy, indicated by statements such as “*I still cannot figure out what to do: if my children are already vaccinated, why should I vaccinate them again”, ”My children have been vaccinated with all the vaccinations, but in the polio case, I really do not know what to do”.*

A telephone survey of a representative sample of 1015 parents of children aged 10 and under revealed clear evidence of hesitant behavior [13]. Parents faced with the request to vaccinate their children with OPV exercised two modes of decision-making. 44 % of the parents declared that they were able to decide immediately on whether to vaccinate their children or not. At the same time, 41 % of the parents claimed that they needed some time for reflection before deciding what to do [13]. Interestingly, the compliance rates with polio vaccination were 82 % among "immediate deciders" and 70 % among "late deciders". These findings suggest that the outcome of vaccine hesitancy is not necessarily non-compliance, and can very often result in a decision to comply with vaccination.

### Vaccination hesitancy related to an emergency H1N1 influenza vaccination

One of the most pronounced manifestations of vaccine hesitancy in Israel occurred during the 2009 outbreak of pandemic H1N1 influenza. This originated from the high level of uncertainties associated with the pandemic. A notable discrepancy was observed between the actual severity of the H1N1-driven disease during 2009 and the fearsome predictions of the professional community. In addition, the H1N1 vaccine, though based on well-established production concepts, was perceived as new and questionable. Moreover, the formulation used in Israel contained a non-traditional adjuvant (squalene) that raised doubts about safety. All these concerns were the subject of a very vivid public debate, which broadcasted the contradictory opinions of “real experts” and “self-proclaimed experts”. This obviously led to disorientation and confusion which provided fertile ground for hesitancy on the part of the public. This hesitancy was rapidly translated into abstention leading to a very low rate of compliance (13 %). A survey conducted to examine the attitudes of the Israeli public to pandemic H1N1 revealed a rather rational risk assessment process, which led individuals to decide against vaccination [14].

### Vaccination hesitancy related to Human Papilloma Virus (HPV) vaccination

Introduction of HPV vaccination programs for 8^th^ grade girls, and more recently for 8^th^ grade boys, led to a considerable public debate in Israel, similar to that observed in other countries. The concerns of Israeli parents were evaluated by focus group discussions and surveys [15]. Parents participating in focus groups discussions raised concerns about the safety of the HPV vaccine, asking weather safety tests in boys were meticulous enough. Concerns were also raised about the effect of vaccination on the sexual conduct of their children and complaints were made about lack of information about the risk of HPV for males [15]. In addition parents have stated that the responsibility for vaccinating their adolescent children is a heavy burden that entails considerable amounts of struggle and hesitancy. This is expressed by one of the participants as follows: “*This cannot be a decision of a 13 years old child. This is the responsibility of a parent. This is a difficult decision. I personally will probably decide to vaccinate, but I don’t know about the others. This is not easy at all*”.

In a recent survey of a representative group of Israelis (*N* = 600), parents were asked about their intentions regarding HPV vaccination of their adolescent sons: 75 % indicated that they intend to vaccinate their sons, 18 % indicated that they don’t intend to do so, and 7 % stated that they have not decided as yet [15]. The last group which can be defined as “HPV vaccine hesitant” is rather small and is similar in size to the group of parents exhibiting hesitancy towards established childhood vaccines (see above). It should be noted that analysis by population groups reveals higher hesitancy among ultra-religious (16 %) and religious (9.5 %) groups, compared to secular Jews and Arabs, suggesting that the reasons driving HPV hesitancy could be different than in the case of established childhood vaccines.

### Vaccination hesitancy related to vaccination of health care workers (HCW)

In Israel, as in other developed countries, health authorities have formulated specific recommendations for vaccination of HCW. Unfortunately, these recommendations are met with only partial compliance. In spite of an extensive campaign, vaccination rates of hospital workers in Israel in 2014 ranged between 10-70 %, depending on the hospital. Likewise, compliance of nurses in Mother and Child Health Centers (MCHC) with recommended vaccines is far from being satisfactory [16]. Attitudes of HCW in Israel to vaccination have been examined in a number of studies [16–18]. Hesitancy of HCW is expressed as a conflict between trusting the recommendations of health authorities and trusting their own expertise [16]; Distrust in the health information provided by their employers versus their perception of risk [17].

## Characteristics of vaccine hesitancy in Israel

Careful analysis of the various manifestations of vaccine hesitancy in Israel reveals several characteristic features that are stated bellow.

### Vaccination hesitancy is associated with higher education and maturity

Vaccination hesitancy appears to be more prevalent among mothers with academic education or older mothers [13, 16, 19]. Moreover, vaccination hesitancy is encountered among nurses dealing with vaccination who are supposed to be knowledgeable about the need to get vaccinated [18]. All this suggests that hesitancy may not be an outcome of ignorance but rather a reflection of the situation where more knowledge generates more doubts.

### Vaccination hesitancy in Israel is culture dependent

There appears to be a substantial difference in the attitude towards vaccination between the Arab and the Jewish populations in Israel. Arabs appear to be more conformist when addressing health requirement and express lower levels of reflexivity and skepticism related to vaccination [7]. Interestingly, the tendency to conform to authorities transcends into high acceptance of a potentially controversial program such as HPV vaccination of boys [15].

### Vaccination hesitancy is about dealing with complexities

In recent years, the Israeli public has faced complex challenges related to vaccination. These have included: dealing with substantial uncertainties associated with the 2009 H1N1 influenza outbreak, dealing with the social responsibility rationale of the 2013 emergency polio vaccination program, and very recently, dealing with the moral implications of vaccinating boys against HPV. The general public was aware of these challenges: Israelis expressed legitimate doubts during the H1N1 pandemic [14], Israeli parents were aware of the fact that they vaccinate their children against polio for the benefit of others [13], and understood that vaccinating boys against HPV protects their female partners from cervical cancer [15]. In all these cases, hesitancy can be viewed as an act of processing complex information and dealing with it.

### Vaccination hesitancy is about making choices

Vaccination in modern societies requires making choices. Individuals are required to choose between the anti-vaccination and pro-vaccination agendas, broadcasted daily by various media sources [11], between the dangers of infections and the "presumed dangers" of vaccines [12–15], and between personal interests and duties to society [15].

### Vaccination hesitancy is about being differential

Vaccination hesitancy is context dependent. Certain programs are met with higher hesitancy than others. This is manifested by positive attitudes towards compliance with routine childhood vaccines yet skeptical attitudes towards seasonal flu vaccination [7]. An additional aspect of differentiation is the low acceptance of the emergency H1N1 vaccination versus the high acceptance of the emergency polio vaccination program [13, 14]. Finally, it is interesting to compare the undisputed acceptance of the newly introduced rotavirus vaccine [20], as opposed to the hesitancy associated with the introduction of HPV vaccination [15].

### Vaccination hesitancy is about self-navigation

Hesitancy is an introversive process, where individuals are concerned with their personal feelings and ideas and try to use this inward examination to navigate their actions. In the case of vaccine hesitancy this could entail an individualistic self-centered evaluation of vaccination programs [7]. In a comprehensive survey (*n* = 2018) of the Israeli population, many respondents stated that vaccination should be left to the personal choice of each individual. This was less noticeable when addressing childhood vaccination (27 % of respondents) and more noticeable when addressing flu vaccination (43 % of respondents). The same motive reemerged in a survey targeting Israeli nurses, where 64 % of the respondents stated that *“Public health nurses should be given full independence to decide whether to receive the recommended vaccine for healthcare workers"* [13]. Another indication for the role of self-navigation in vaccine acceptance is the observation that a low external focus of control can serve as a predictor for failure of parents to comply with childhood vaccination programs [8], suggesting that non-willingness to defer to others is a constituent of vaccine hesitancy.

### Vaccination hesitancy is about judicious trust in authorities

Hesitancy is contradictory to non-conditional trust in health authorities. Nevertheless, hesitancy does not necessarily mean full rejections of vaccine related recommendations. Examination of the reaction of the Israeli public to pandemic H1N1 influenza vaccination revealed a complex interrelationship between trust and compliance. A large fraction of the population (35 % of respondents in a targeted survey) exhibited “trusting non-compliance”, namely trust in authorities accompanied by a decision not to get vaccinated [21]. A different profile was observed in the case of the 2013 emergency polio vaccination campaign, where trust coincided with the belief that vaccination is justified [13].

### Vaccination hesitancy is about learning and acquiring knowledge

The time-lag associated with hesitancy is often used to gather information. One such example relates to parents who delayed their decision during the emergency polio vaccination in order to learn more. The information sources used by these parents included: health care professionals (72 %), friends and family (70 %), the media (69 %), MOH mediated information (59 %) and the internet (66 %). It should be noted that 38 % of the deliberating parents used 3–4 different sources and 31 % used as many as 5 information sources [13].

### Vaccination hesitancy is about assessment and argumentation

The learning process associated with hesitancy leads most often to an assessment process [13, 14] where individuals balance the pros and cons of getting vaccinated. This involves evaluation of the danger of the infectious disease at question, the risks of getting infected, the potential risks of the vaccine, and its perceived efficiency. This evaluation leads eventually to a decision which is not necessarily the right one in terms of personal safety and public interest, but is still the result of a personal deliberation. It is interesting to note that, when interviewed about their decisions, interviewees tend to provide solid arguments to justify their decisions [13, 14]. Rationalized argumentation can also be identified in online discussions amongst lay individuals [11, 12].

## Conclusions

Taken together, all the characteristics described above suggest that vaccine hesitancy is not necessarily a manifestation of passiveness or feebleness. On the contrary, hesitancy can be a very proactive process. Lay individuals choose not to rely on authoritative agencies, such as the medical establishment, nor on alternative agencies such as the anti-vaccination movements. Instead, individuals make their own decisions in a process which involves assessing, choosing and dealing with complexity. This appears to be an act of self-determination, where individuals exercise reflexivity and self-empowerment when deciding about vaccination.

In Israel, the manifestations of reflective vaccine hesitancy appear to rely on two traditions: the long-lasting Jewish scholarly tradition, and the more recent socio-political tradition of Modern Israel. On one hand, the process of learning in the Jewish tradition employed, for centuries, mechanisms of debating, arguing and questioning [22], which resemble some of the hesitancy mechanisms described above. On the other hand the short history of the State of Israel is marked by quarrels, social schisms, divergence of opinions, distrust of authority and contempt of leadership [23]. This could serve as a fertile ground for an atmosphere of skepticism towards the requirements of medical authorities, where vaccination hesitancy is enhanced.

While these local effects appear to play a notable part in the evolvement of vaccine hesitancy in Israel, one cannot overlook worldwide changes that contribute to this trend. These include shifts in the attitude towards vaccination in general [24, 25] together with growing tendencies to promote patient empowerment and encourage lay individuals to govern their personal health. All this is embedded in other manifestations of late modernity, such as a shift in responsibility from State agencies to individuals, and realization that that coping with risk requires a continuous process of evaluation, reassessment, reorientation [26]. Thus, vaccine hesitancy as an act of self-determination is most probably not confined to Israel.

The proper definition of vaccine hesitancy is essential for designing vaccine-promotion policies. One can portray vaccine hesitancy as a broad spectrum of phenomena, ranging from a genuine call for help to complete defiance of authorities. In this article, emphasis was made on mid-spectrum hesitancy; hesitancy as an act of exploration and self determination. Focusing on this aspect is not only very relevant to the Israeli scene but could also be instrumental when addressing hesitancy. Dealing with individuals who are genuinely asking for guidance is relatively easy, while dealing with very defiant individuals can be futile. In contrast, dealing with mid-scale hesitant individuals that are struggling with vaccination could be challenging, but ultimately fruitful. This requires designing appropriate intervention strategies but also a change of attitudes.

The first step should involve realization and adaption. Policymakers should realize that hesitancy is an act of self-empowerment, which can result in good decisions, but also in very dangerous decisions. They can resent this and oppose this, but they cannot ignore it; deliberative hesitancy is here to stay. The second step should involve development of respect towards hesitant individuals. A parent worried about the potential deleterious effects of vaccination should be respected as a responsible person, even if his fears are not grounded. A lay individual trying to gain knowledge prior to committing to vaccination should be respected, even if his ability to process the information is imperfect. A person deciding not to comply with vaccination after a long process of deliberation should be respected, even if her decision is faulty. Once respect is embedded in the attitude of health professionals, one can start paving the way to interacting, explaining and convincing the hesitant public about the advantages of vaccination.
